# Report of the Demonstrator of Operative Dentistry of the G. V. Black Dental Club (Inc.), of St. Paul, for the October, 1903, Meeting

**Published:** 1904-01

**Authors:** E. K. Wedelstaedt

**Affiliations:** Demonstrator


### REPORT OF THE DEMONSTRATOR OF OPERATIVE DENTISTRY OF THE G. V. BLACK DENTAL CLUB (INC.), OF ST. PAUL, FOR THE OCTOBER, 1903, MEETING.

BY E. K. WEDELSTAEDT, DEMONSTRATOR.

Mr. President and Gentlemen,—For a number of years you have elected me to the office of demonstrator of operative dentistry. Each month during this long period of time a report has been made you regarding the current dental literature of the day and some special features of our operations. There has also been a large number of discussions of the ideas and methods advanced by others. On many occasions we have wondered at some of the things printed in the journals, and many times we have been unable to understand how it was possible for men to display such a limited comprehension of our operations.

In my dealings with you the changes which have taken place in your ideas regarding our methods and operations have perhaps been of more satisfaction to me than anybody knows. This continual advance is as it should be. You know that intelligent men who are interested in their different vocations do more or less reading, as well as have a constant interchange of ideas with others in their special calling. By following such a course, we change our ideas, our methods, our operations, etc. In the fewest possible words, we broaden our views and look at things generally in a much more comprehensive way. A man who does not do any reading cares nothing for an interchange of opinion with others, is perfectly satisfied with himself and his methods, is not, in fact, interested in what he himself or others are doing; he becomes more and more narrow, until at last his ideas may not be broader than the edge of a razor. It is from the men who are in the last-named class that we hear so much about the beauty of following the obsolete methods. As a rule, these men are unwilling to progress, and, worse still, are unwilling to permit others or the profession to advance beyond their own ideals or standards. Imagine for a moment the situation, if other professions were handicapped in a similar manner. Suppose the medical journals were continually filled with many essays which should advocate the necessity for bleeding all people who had fevers. Or suppose, at the regular meetings of physicians, there was a constant discussion regarding the efficacy of following the hot-water treatment of Dr. Ferrara, so beautifully depicted in Gil Blas, what advance in their calling would physicians make? To speak plainly, we have altogether too many writers who are forever dwelling on the past history of our profession and who are forever giving entirely too little consideration to the present and future welfare of our calling. No man, I do not care who he is, honors the memory of Townsend, Varney, Webb, Cushing, McKellops, and all others who were instrumental in assisting to raise the standard of our profession, more than I do, nor does any man place a higher value upon their efforts in doing good. But I also try to make use of the knowledge which has been developed since the time of these men, instead of continually dwelling on the beauty of the ideas that were promulgated twenty or thirty years ago. It is hardly necessary for me to say to you, that unless I could assist in raising the standard to a higher plane, try to interest you in progressive dentistry, try to interest you in being of greater worth to humanity, both my pen and voice would be forever silent, and I would not, month after month, come to you as I have requesting that you make greater use of and give more consideration to the knowledge as it has been developed. An honest dentist is the noblest work of God, and an honest dentist dwells rather with the present than with the past. He takes the best out of the old and applies it to the new. He is a radical, but he is the king of conservatives. Intelligent men so recognize him and the position which he has taken, but ignorant men are apt to call him an extremist. Few persons seem to know that the term "extremist" is equally applicable to ignorant as well as to intelligent people. You know also that the greater the departure from our own personal standard or ideals the more we are inclined to think it extreme.

But all this preface has been for the purpose of leading up to other things.

On many occasions we have all wondered how long it would be before some little ray of light would begin to break in on others, and when they would look at conditions from our point of vantage. The light has at last reached one of my friends. In the INTERNATIONAL DENTAL JOURNAL for September is an essay from the pen of Dr. M. L. Rhein, of New York. The subject chosen is, "The Technique of Approximal Restorations with Gold in Posterior Teeth." The essay, being published, is public property, and I have a right to discuss the ideas which it contains. The essay has been read by me a number of times, and I shall try to discuss it in a perfectly fair and impartial manner. To discuss it in this way, however, is a somewhat difficult thing to do, for the reason that there are so many unknown terms used with which I am not familiar. Neither can I quite understand the use of a number of words for which there is no authority and which, so to speak, do not quite fill the bill. Besides, Dr. Rhein's interpretation of the fundamental principles is very different from our own, he being a follower of Webb, while we are followers of Black. (It may be well to state that Webb died in 1882.) On account of making use of the newer knowledge which has been developed during the past twenty years, as well as of the experience gained during these years, a somewhat different interpretation is now given the fundamental principles from those which were given them at the time of Webb. The results of the experiments of the physical properties of the human teeth and filling-materials, the development of our knowledge of stress and its influence on our operations, the development of bacteriology, the setting at rest of the cause of decay of the human teeth, the classification and use of remedial agents, a continual development of our knowledge of the cause of failure of fillings and the conditions of faulty environment, the development of principles of instrumentation, etc., are all factors which have tended to give us a very different interpretation of the essentials from those that we once possessed. But just so long, however, as men are unwilling to make use of the knowledge spoken of, which in altogether too many cases they will not even consider, there will not be harmony among us in so far as this relates to a better understanding of what is necessary. As we make use of the knowledge gained from a constant study of the conditions with which we deal, so are we able to grasp much better our hold on the fundamental motives for every step of our operations. The profession of dentistry cannot advance until these different things are considered and studied as they should be.

What I wish above all else is that we could persuade Dr. Rhein to spend about a week with the mighty Searl. I have an idea that at the end of this time he would be perfectly satisfied with the knowledge that to be a follower of Black was about the greatest honor man could attain in an ordinary lifetime. The good thereafter which Dr. Rhein would be capable of rendering advanced dentistry would be of incalculable value. But let us to his ideas.

Dr. Rhein has at last recognized the value of having fillings properly seated on a flat gingival wall or seat. From this time forth he will begin to have much satisfaction from noting the stability of the beautiful operations which he is capable of making. The necessity for squaring out the lingual and buccal gingival angles is nicely considered. He recognizes the uselessness of making large and deep undercuts, for these merely weaken the tooth and do not add stability to the filling. The illustrations published in connection with his essay do not, by any means, give us a clear conception of his ideas in regard to an occlusal anchorage. His words, however, cannot be misunderstood when he speaks of the value of right-angle anchorages in the occlusal surface. There is a difference in our methods of cavity preparation, but it is too slight to speak of, with the exception of his treatment of the cavo-surface angle. He trims this angle with a gem stone and then finishes it with sand-paper disks, a method I have observed many men use. Tight margins cannot under any circumstances be made against a smooth margin if gold is the filling-material used. Gold slides over a polished surface, for there is nothing against which it can be restrained in its course. But roughen that margin by planing it with a sharp chisel, and gold will hug that roughened surface, if properly placed, so that water-tight margins can be made. Dr. Rhein, among other things, does not believe in the use of soft or unannealed gold in the gingival third of cavities which we fill in the proximal surfaces of molars and bicuspids. He believes in an all-cohesive gold filling. He anchors his first piece of gold with a small quantity of cement at the junction of the axial wall with the gingival seat,—*i.e.*, gingivo-axial angle. (Dr. Rhein has not been very explicit in the use of terms. I do wish that he had been, so that we could have better understood what he means.) He also believes that after the gingival third of the cavity is filled the filling can be polished; that the matrix can be used; that the gold in the proximal surface should be entirely built up to the step on the occlusal surface; that the electric mallet will pack gold to homogeneous compactness; that those belonging to the Black creed write as though all recurrence of decay is due to non-extension of cavity margins; that we can stop packing our gold into cavities at any point and any number of times, fill the cavity with gutta-percha, and at a subsequent sitting cut down the gold and go on with the operation; that after the cavity is ready for the gold it should be washed with a few drops of ten per cent. solution of formalin, etc. It will not be possible for me to consider these nine subjects in any other than the briefest manner.

Let us, for a moment, look into this matter of using soft or unannealed gold in the gingival third of cavities in the proximal surface of molars and bicuspids. The most difficult operation a skilled dentist is called upon to perform is the removal of serumal calculus. Following this operation very closely is the ability to make tight margins with cohesive gold, and there are not very many dentists in our profession who can do either perfectly. Any man who has the ability to make a water-tight filling with cohesive gold in one of the proximal surfaces in molars or bicuspids has my unqualified respect and admiration. It is an operation that I would not under any circumstances attempt to make, for I know of the difficulties with which I have to cope. Let me watch an operator place and condense his gold, and I can very quickly tell you whether or not he is making a water-tight filling. On page 526 of the *Dental Cosmos* for 1891 is said all that is necessary about this subject; besides this, I have given you demonstration after demonstration with gold itself. I wish to say that any intelligent person who will watch and make a careful study of the action of cohesive gold under the plugger-point while being packed into a given cavity cannot help becoming impressed with the difficulty of making tight margins with such an intractable material. If we have all this trouble when filling cavities in the mesial surfaces of the upper incisors and cuspids, where conditions are most favorable to success, what can we expect when we deal with cavities in the mesio- or disto-occlusal surfaces of molars and bicuspids? Dr. Rhein has, however, good reasons for his contention. I notice a number of essays in our journals in which the writers advance the idea that a matrix can be placed around a cavity and the whole proximal surface be filled with soft or unannealed gold. Now this is a very common mistake, and it is being made by too many operators. Because a few men advance ideas of this kind is not, in my estimation, a good reason for condemning entirely the use of soft or unannealed gold. Let us take a practical case. There is a cavity to be filled in the lower left first bicuspid. The linguo-bucco-gingival measurement of the cavity is five millimetres. The gingivo-occlusal measurement is 4.5 millimetres. The gingival seat is 1.7 millimetres. The man closes the gnatho-dynamometer to the one-hundred-and-fifty-pound mark. The filling will have an occlusal anchorage. I will take three cylinders made of one-quarter of a sheet of No. 4, untrimmed gold and place them in the gingival third of this cavity and then condense them. On remeasuring the cavity I find that those three cylinders have reduced the gingivo-occlusal size of the cavity 1.5 millimetres. Now, when the cavity is filled the cohesive gold will be 3.5 millimetres thick. This will be amply sufficient for all practical purposes. I cannot conceive what objection there would be to the use of this small amount of soft or unannealed gold. Further, I feel very certain in saying two things: First, that those three cylinders could be placed and condensed before any person could ever mix cement; secondly, that ninety-nine per cent. of operators are more likely to make tighter margins by using soft or unannealed gold in the position named than could ever be made by the use of cohesive gold.

Where patient after patient consults me, in whose teeth I find fillings which have had the gingival third of the cavities in molars and bicuspids filled with soft or unannealed gold, that have done good service for upward of thirty or more years, I am perfectly willing to continue to use it, especially when I am continually seeing failures of fillings which have been made with all-cohesive gold. Several years ago a long line of experiments with cements were published. Since that time these results have been added to by others who have also made experiments. Now let us consider this matter a little farther, and meet it fairly as men should do. At the present time we have on the market and in constant use cements which are water-logged within two hours after being placed in the cavities in the human teeth; cements which contract and cements which expand. We know definitely that it takes fully five days for cements to obtain their full degree of hardness and settle down into an inert condition. This being the case, I cannot understand how Dr. Rhein can advocate any such method of anchoring his first piece of cohesive gold. Another thing. For a number of years I have removed many gold fillings which have had the first piece or pieces of gold anchored as described by the essayist. In fact, since beginning the writing of this report I have removed three gold fillings which have been anchored according to this method. The cement in these three cases was simply a white powder without structure or form, and this is precisely as I have always found it where this method has been employed. In view of these circumstances, it is deplorable that others are asked to follow a method the ultimate results of which are very far from satisfactory.

Polishing the gold placed in the gingival third of cavites before the entire filling has been made.

Filling part of the cavity with gold and leaving it to be finished; *i.e.,* to continue the placing of gold at another sitting.

Using a few drops of ten per cent. formalin just prior to placing the gold into the cavity.

Anybody who has made experiments with gold fillings knows that the best operations are made where gold is placed against a perfectly clean surface. A clean, freshly roughened surface, such as is made with a sharp chisel, is the only surface gold will hug closely. In fact, it is the only surface against which we can pack gold and make water-tight margins. With this knowledge before us, how is it possible to finish the gold which has been placed in the gingival third, polish it with strips which have been vaselined, and not be compelled thereafter to go over the remaining cavo-surface angle with a sharp chisel. This must be done. Besides this, there will be a bad joint where the filling is continued. Nor is this all. There is a fundamental principle involved which demands some consideration from us, and it is the law governing marginal condensation. If we believe in marginal condensation it is impossible to make a filling of homogeneous compactness in the gingival third only, for the reason that in making the marginal condensation the gold towards the occlusal surface, not being supported, is stretched and driven from its position. How it is anchored in the cavity does not make any difference. It is impossible not to drive the gold in the direction of the least resistance. The gold, not being supported towards the occlusal surface, is spread in that direction to the detriment of the rest of the filling. By following such a method we induce lines of internal weakness in the filling itself. No, I cannot endorse a method of this kind.

After a cavity is once finally prepared, the gold should be placed then and there, or else we should cut a new cavo-surface angle prior to placing our filling-material. And after the cavity is prepared and ready for the gold, no medicine of any nature should ever be placed therein (except, occasionally, when a little varnish may be used on an axial or pulpal wall). When the cavity is finally prepared it is in the best possible condition to make water-tight margins, therefore fill it. I cannot understand the notion possessed by so many operators that all cavities should be washed with alcohol, chloroform, oil of cloves, carbolic acid, or some other agent just prior to placing the filling-material. I view the use of such methods with many misgivings, so far as they relate to the final results.

We have all tried and know what results are obtained where a matrix is used, so far as this relates to making gold fillings. If there is a place in dentistry where they are contra-indicated it is in connection with the use of gold. The matrix and amalgam go hand in hand together, and there let the matter rest. In the fewest possible words, matrices have absolutely no place, nor are they useful adjuncts to employ where we are dealing with gold.

In placing gold in the cavities in the proximal surfaces the best results are obtained if the gold is laminated from the anchorage in the occlusal or incisal surface towards the gingival seat. Surely far stronger fillings can be made by following such methods than by first filling the entire cavity in the proximal surface up to the anchorage in the occlusal or incisal surface. The essayist's remark about keeping gold about even is what has been taught for a number of years and his emphasizing this teaching is to be commended.

The essayist says, "In fact, all the members of the Black creed write as though all the recurrences of decay that are found are due to insufficient extension." Now this statement is to be regretted. You gentlemen are intelligent men, and I am very glad that we never hear anything about extending cavity margins. For nearly two years not a man in this Club has said anything to me about applying principles of extension for prevention. This continual harping on this subject is absolutely disgusting. When surgeons meet they do not continually discuss the necessity for sterilizing their instruments, neither are the essays in the medical journals continually dwelling on this one topic. All rational surgeons recognize the necessity for sterilizing their instruments. All rational dentists, who have made a study of their failures, recognize the necessity for the application of principles of extension for prevention. Extension for prevention is simply one of the essentials which it is necessary to consider when we deal with cavities that are in many proximal surfaces. But there are other things besides applying principles of extension for prevention, and these other things call for our most earnest consideration. Cavities at all times must be made accessible, fillings must be properly seated and anchored, gold must be properly placed and condensed, fillings must be trimmed to form and polished, the tooth must be returned to its original mesio-distal diameter, and there must not be a condition of faulty environment left. Each of these subjects is of equal importance, and goes hand in hand with the application of principles of extension for prevention. Ignore one of them, and later on failure will stare you in the face. It was only a short time ago that a patient consulted me regarding the condition of her teeth. The fillings were most beautifully made, trimmed, and polished. The cavities in the proximal surfaces, on account of being refilled so many times, were three-quarters as broad linguo-buccally as the teeth were thick, but there was no contour given the interproximal space, nor could it be done because the teeth were not returned to their original mesio-distal diameter. Within three years these fillings will have to be remade, as there is no possibility on the part of the patient of keeping them clean. No operator, I do not care who he may be, can leave a condition of faulty environment surrounding any dental or surgical operation and expect to obtain anything else than failure. I hope that so long as I am a member of this Club we shall never again be called upon to hear anything about the necessity for the application of the principles of extension for prevention.

There remains, then, but one subject to discuss, and that is the use of the electric mallet in condensing gold. Like many other operators, I formerly used the electric mallet. For a number of months it was in constant use in my office, when it was discontinued. My reasons for this were simply that I could operate more rapidly by other methods and make much better operations in a shorter space of time by employing an assistant who used a hand mallet. Along in 1895 some tests were made with gold fillings which had been made by using different mallets, different kinds of gold, etc. In the *Dental Cosmos* for that year, on page 749, can be found the results of these tests. An expert in the use of the electric mallet made three fillings for me. The highest specific gravity reached was 13.2. From an examination of the results obtained by testing the different fillings, we find that the electric mallet is not even on a par with the automatic mallet, and until Dr. Rhein or some other good man will make us some experimental gold fillings, using the electric mallet for packing his gold, and give us the benefit of the results of his tests, the results of the experiments in the 1895 *Cosmos* must be considered standard. I think that Dr. Rhein may be able to make fillings of 16 or more specific gravity by using the electric mallet, but it will be necessary for him to give each piece of gold condensed from three to five minutes malleting. When it is taken into consideration that twenty seconds of hand malleting on each piece of gold, of a given and known size, will give a specific gravity of 17 or more, are we justified in taking a longer time? Are not our operations long enough, and are they not tedious enough?

I have taken this essay and have made an attempt to discuss it in a way that was absolutely free from all prejudice. Attention has been called to a number of things, and they have been discussed as thoroughly as it has been possible for me to do in such odd moments as I could obtain from my labors. When it is known that a man of Dr. Rhein's standing will make changes in his methods to the extent he has, there is hope that others will do likewise.

I stand for a constant advance in our chosen calling. I have made a few contentions for that which I considered was of value in advancing dentistry to the position where I feel it should be. That I have made mistakes nobody knows better than I. Had I not made and recognized them, I could not have discussed this essay in the way I have.

I suppose that you wish me to say something about the gold fillings which we examined this morning. The operations were made by Dr. J. V. Conzett, of Dubuque, Iowa. In the past twenty-five years I have never seen eleven more beautiful operations in the teeth of the same individual which had been made in cavities in the proximal surface of molars and bicuspids. In each operation the cavity margins had been placed in the most convenient position for making as nearly perfect operations as possible. The contour of the interproximal space was as perfect as it was possible to obtain. The contact points were small and correctly placed, with the exception of those made on the fillings in the proximal surfaces of the upper left first molar and second bicuspid. The contact points on these two fillings had been placed a trifle too far towards the disto-bucco and mesio-bucco-occlusal angle, but these two fillings have not been completely finished. At the final finishing it may be possible to alter the position of those two contact points. This is the only comment I have to offer regarding the operations. The knowledge that we have men in this Northwest who have the ability to make such operations and do make them, is an incentive to our ambition. Not only is it a revelation to know that we have men who have ideals and are perfectly willing to carry them out, but examining such operations aids us materially in an educational way, by stimulating us to a higher endeavor. This is the kind of dentistry that I have been trying to interest men in for the last eight years, and I am perfectly satisfied with the results. I cannot speak too highly of the benefits that accrue to a patient who has such operations made in the cavities of his teeth. I have been particularly interested in Dr. Conzett and what he is doing. For the past five years he has given up much time to a study of conditions, in seeking out the cause of his failures, and in having an interchange of opinion with other men in our calling. We are now in a position to judge of his interpretation of the fundamental principles and how he applies his knowledge to the operations which he makes for others. Comparisons are at all times odious, but right in connection with this subject I desire to present for your consideration these two casts. The fillings as outlined in that molar and bicuspid were made some eighteen months ago by a man of national reputation and a teacher of operative dentistry in one of our colleges. As Dr. Searl so nicely says, "The operations made by Dr. Conzett were made to preserve the teeth, and those made by the teacher were made for what money he could get. They were not made to preserve the teeth." The patient was thirty years of age, and he was one of the best patients for whom I have ever operated. Whether "skipping corners" in this way, as Dr. Black says, calls for a display of charity is something which you must decide for yourselves. In the operations made by the teacher every fundamental principle was ignored, so far as this relates to those gold fillings. In the operations made by Dr. Conzett we have the application of every known fundamental principle. On this account the operations do not compare very favorably one with the other. Dr. Conzett is an honest dentist. Where to place the teacher, I do not know nor do I wish to know.

All of which is most respectfully submitted.

